# Advances in donor-derived cell-free DNA monitoring for solid organ transplantation

**DOI:** 10.3389/fimmu.2026.1769036

**Published:** 2026-03-06

**Authors:** Darius Sairafi, Emelie Foord, Linnéa Pettersson, Michael Uhlin

**Affiliations:** 1 Devyser AB, Stockholm, Sweden; 2 Department of Medicine Huddinge, Karolinska Institutet, Stockholm, Sweden

**Keywords:** allograft rejection, assay standardization, biomarker, digital PCR, donor-derived cell-free DNA, next-generation sequencing, transplantation

## Abstract

Donor-derived cell-free DNA (dd-cfDNA) has emerged as a minimally invasive biomarker of allograft injury following solid organ transplantation. However, its clinical performance and interpretability depend strongly on how dd-cfDNA is measured, reported, and integrated into existing care pathways. This narrative review outlines the biological rationale for dd-cfDNA monitoring and explores the key preanalytical and analytical factors affecting test performance, with an emphasis on comparing measurement technologies and commonly used diagnostic systems. We examine major assay strategies, including next-generation sequencing approaches and PCR-based methods, including digital PCR, and discuss how assay design influences the need for donor/recipient genotyping, analytical sensitivity, susceptibility to clinical confounders (e.g., early post-operative injury, infection, leukopenia, and multi-organ DNA sources), turnaround time, batching, and quality control requirements in centralized versus decentralized testing models. We synthesize evidence for clinical validity and utility across transplanted organs, focusing on use cases such as early detection of injury, risk stratification, and supporting biopsy decisions, while highlighting ongoing challenges like threshold harmonization, inter-platform comparability, and imperfect specificity for distinguishing rejection from non-rejection injury. Overall, dd-cfDNA serves as a valuable adjunct for graft surveillance, but broader clinical applications will require standardized guidance for assay performance and reporting, platform-aware interpretation frameworks, and prospective outcome-focused studies to define optimal testing intervals and decision thresholds.

## 1 Introduction

Traditional monitoring after solid-organ transplantation relies on clinical assessment, functional tests, and tissue biopsies. While biopsies provide histological evidence of graft status, they involve procedural risks, sampling errors, interpretation variability, patient discomfort and high costs. Moreover, by the time functional markers indicate problems or biopsies are performed, significant allograft damage may have already occurred. This highlights the need for non-invasive tests and sensitive biomarkers capable of early detection of allograft injury (1). Detection of donor-derived cell-free DNA (dd-cfDNA) in recipient blood has emerged as a promising non-invasive biomarker for monitoring allograft health. Elevated dd-cfDNA can be detected before conventional signs of graft dysfunction appear, enabling early intervention. Advanced molecular techniques now allow for the precise quantification of donor DNA within recipient cfDNA (2, 3).

This review provides an overview of the progress in dd-cfDNA monitoring in solid organ transplantation, focusing on technological advances and clinical applications. It covers the technological foundations, exemplifies clinical validation and performance across various organs, economic aspects, and future prospects of dd-cfDNA monitoring for personalizing post-transplant care. Relevant publications were identified through a non-systematic search of the peer-reviewed literature Pubmed/MEDLINE and the Cochrane Library, last updated in October 2025. Given the rapid evolution of dd-cfDNA technologies, we focused primarily on studies published between 2015 and 2025, while also including seminal earlier publications where needed for methodological or biological context. Consistent with the narrative nature of this review, study selection did not follow a prespecified systematic protocol; instead, publications were prioritized based on relevance to analytical methodology, clinical validation, and reported clinical utility across solid-organ transplant settings. We also specifically prioritized studies evaluating commercially available dd-cfDNA assays in current clinical use.

### 1.1 Biology and significance of donor-derived cfDNA in allograft health

Dd-cfDNA consists of DNA fragments released into the recipient’s bloodstream from the transplanted organ, primarily due to apoptosis and, to a lesser extent, necrosis during acute injury (4). These cfDNA fragments are typically short (160–180 base pairs), reflecting nucleosomal patterns resulting from apoptotic DNA cleavage (5). Due to its short half-life, ranging from 30 min to a few hours, dd-cfDNA levels dynamically reflect the current status of the allograft (6, 7). In a stable and well-functioning allograft, there is a baseline level of cellular turnover, resulting in minimal release of dd-cfDNA. The exact baseline fraction of dd-cfDNA differs depending on the graft type, with larger organs, such as the liver, typically contributing to a higher percentage than smaller organs, such as the heart or kidney (2, 8). Following transplantation, dd-cfDNA is initially elevated in response to surgical injury and ischemia-reperfusion stress before it declines to organ-specific baseline levels (7). The clinical significance of dd-cfDNA lies in its function as a direct, non-invasive marker of allograft injury. Various pathological processes affecting transplanted organs, such as acute rejection (including T cell-mediated rejection (TCMR) and antibody-mediated rejection (AMR)), chronic rejection, infection, ischemia-reperfusion injury, and drug toxicity, can lead to increased cell death within the allograft. This, in turn, results in a measurable increase in the amount of dd-cfDNA released into the recipient’s bloodstream (1, 9). Numerous studies have demonstrated that elevated dd-cfDNA levels are associated with biopsy-confirmed rejection and other types of graft injury across various solid organ transplants (10–12). Notably, increases in dd-cfDNA can often precede the appearance of clinical symptoms or changes in traditional functional biomarkers, enabling the detection of subclinical graft injury (13). This early detection capability is crucial, as it may allow for preemptive or early therapeutic intervention, potentially reducing the severity of the injury, preventing progression to chronic damage, and ultimately enhancing long-term allograft survival. Additionally, low levels of dd-cfDNA can offer a high negative predictive value (NPV) for active rejection, potentially aiding in avoiding unnecessary invasive biopsies in stable patients post-transplantation. The dynamic nature of dd-cfDNA also facilitates monitoring of the response to treatment as a decrease in dd-cfDNA levels following anti-rejection therapy can indicate successful resolution of graft injury (7). The reported cut-offs for distinguishing acute rejection or its subcategories vary, but a 1% threshold is commonly applied for kidney and lung, while a 0.2% threshold is used for heart. For liver, a higher cut-off, generally around 10%, is used, as further discussed later (**Table 1**).

**Table 1:** Selected key studies of donor-derived cell-free DNA (dd-cfDNA) across solid-organ transplant types. Studies are grouped by transplanted organ and summarize reported diagnostic performance alongside the underlying measurement methodologies and representative commercial dd-cfDNA assays.

Study/platform	Study details	Cut-off	Performance parameters	Key notes from study	Reference
Kidney					
Bloom et al., 2017 NGS; AlloSure (CareDx)	Multicenter prospective (DART) study with 102 pts and 107 biopsy−matched samples (80 no-AR and 27 AR; 16 ABMR, 11 TCMR)	1.0%	Creatinine: AUC 0.54 AR: AUC 0.74; Sens 59.3%; Spec 84.7%; NPV 84%; PPV 61% ABMR: AUC 0.87; Sens 81.2%; Spec 83.1%; NPV 96%; PPV 44%	Dd-cfDNA outperformed serum creatinine; dd-cfDNA was also able to discriminate ABMR vs no ABMR; establishment of 1% cut-off. Clinical validation of the first NGS-based centralized dd-cfDNA assay.	(14)
Oellerich et al., 2019 ddPCR; VitaGraft (Oncocyte)	Single-center prospective study with 189 pts (97 for performance; 15 BPR, 83 stable)	0.43% vs 52 cp/mL	BPR (cp/mL): AUC 0.83; Sens 73%; Spec 73%; PPV 13%; NPV 98% BPR (%): AUC 0.73; Sens 73%; Spec 69%; PPV 12%; NPV 98%	Two dd-cfDNA comparators (relative fraction vs absolute quantification); recommended cut-offs 0.5% and/or 50 cp/mL. Clinical validation of the first ddPCR-based centralized assay.	(15)
Halloran et al., 2022 NGS; Prospera (Natera)	Multicenter prospective (TRIFECTA) study with 367 pts (218 for performance); AR vs no-AR by MMDx or histology	1% and 78 cp/mL	MMDx AR: AUC 0.88; Sens 83%; Spec 81%, PPV 68%, NPV 91% Histo AR: AUC 0.82; Sens 74%; Spec 81%; PPV 71%; NPV 83%	Development of two-threshold algorithm using both fraction and quantity; evaluation of histology vs MMDx as comparator.	(16)
Bu et al., 2022 NGS; AlloSure (CareDx)	Multicenter, observational real-world (ADMIRAL) study with 1092 pts and serial dd-cfDNA sampling; for performance 203 pts with 219 biopsies (113 AR; 75 ABMR, 38 TCMR)	0.5 vs 1.0%	AR: AUC 0.798 (creatinine AUC 0.492) *At 1% cut-off:* AR: Sens 58%; Spec 82%; PPV 54%; NPV 84% ABMR: Sens 65%; Spec 82%; PPV 54%; NPV 84% TCMR: Sens 45%; Spec 63%; PPV 31%; NPV 76%	Confirmed ≥1% dd-cfDNA threshold and also demonstrated relative-change threshold of 61% in a real-world setting.	(17)
Aubert et al., 2024 NGS; AlloSure or AlloSeq (CareDx)	Multicenter observational population-based study with 2882 pts (1134 pts as derivation cohort with 1415 biopsy−matched samples and 1748 pts as validation cohort)	Continuous or fixed at 0.5% and 1.0%	*Derivation cohort:* SOC: AUC 0.77 SOC with dd-cfDNA: AUC 0.821 *Validation cohort:* SOC: AUC 0.743 SOC with dd-cfDNA: AUC 0.842	Large, contemporary validation comparing integration of dd-cfDNA to SOC prediction models. Dd-cfDNA improved discrimination of rejection, supporting its use as part of routine surveillance. Highlighted the necessity to combine into an integrative score.	(18)
Pettersson et al., 2024 NGS; One Lambda Devyser Accept cfDNA (Devyser)	Single-center retrospective study with 154 clinical and artificial samples; clinical samples from 31 pts who had undergone two sequential kidney transplantations	N/A	Detection of dd-cfDNA from the first transplanted (non-functioning) graft in 20% of the analyzed patients. Amount of dd-cfDNA detected from the first graft ranged 0.1%–0.6%.	Study on multiple donor setting, a scenario where most available assays are not validated, providing utility in complex multi-donor graft settings.	(19)
Heart					
De Vlaminck et al., 2014 SNP-based shotgun NGS	Two-center prospective study with 65 pts (incl 21 pediatric), 356 EMB-samples; 185 quiescence (0), 147 mild rejection (1R/1A≤grade<2R/3A), 24 mod-to-sev rej (≥2R/3A or AMR)	0.25%	Moderate-to-severe rejection (≥2R/3A or AMR) vs non-rejecting (grade 0): AUC 0.83; Sens 58%; Spec 93% Also reporting on distinguishing moderate-to-severe and mild rejection events as well as other categories.	Early study demonstrating the utility of dd-cfDNA post-heart tx and the potential to improve existing biopsy-based surveillance approaches.	(20)
Khush et al., 2019 NGS; AlloSure Heart (CareDx)	Multicenter prospective (D-OAR) study with 740 pts (443 pts with 841 EMB-matched samples; 35 AR of which 18 AMR and 17 ACR, 806 no-AR)	0.2%	AR: AUC 0.64; Sens 44%; Spec 80%; PPV 8.9% NPV 97% AMR: PPV 4.2%; NPV 98.6% ACR: PPV 4.8%; NPV 98.6% Also reporting per biopsy type (protocol/for-cause)	Median 0.07% in ref samples and 0.17% in AR-classified samples. High NPV demonstrated reliable rule-out capability, supporting reduced biopsy use in clinically stable heart tx-patients.	(11)
Agbor-Enoh et al., 2021 SNP-based shotgun seq	Multicenter prospective (GRAfT) study with 171 pts and 1392 EMB; 749 no rej, 570 mild rej (ACR 1, AMR 0) and 73 AR. 1141 concurrent dd-cfDNA	0.25% (also 0.1% and 0.5%)	*At 0.25% cut-off:* AR: AUC 0.92; Sens 81%; Spec 85%; PPV 19.6%; NPV 99% AMR: AUC 0.95; Sens 88%; Spec 85% ACR: AUC 0.89; Sens 79%; Spec 85%	Providing evidence for dd-cfDNA as a biomarker of cardiac allograft injury from d28. Levels were higher in AMR vs ACR, increased with rejection severity and, in majority, increased months before EMB changes.	(21)
Kim et al., 2022 NGS; Prospera Heart (Natera)	Multicenter prospective (DEDUCE) study with 223 pts, 811 EMB-matched samples; 762 no-AR and 49 AR (32 AMR, 17 ACR)	0.15% (0.12%, 0.20%) *Post hoc:* 13 cp/mL	*At 0.15% cut-off:* AR: AUC 0.86; Sens 78.5%, Spec 76.9%; PPV 25.1%; NPV 97.3% *Post-hoc analysis on absolute quantity:* AR: AUC 0.88	Validation of centralized assay with performance reporting at various thresholds; *post-hoc* analysis using dd-cfDNA quantity indicating that incorporation could increase sensitivity.	(22)
Böhmer et al., 2025 ddPCR	Multicenter prospective study with 94 pts (incl 24 children), 1007 EMB and blood samples; 32 rejection (ACR2/3R and AMR), 885 non-rejection. In performance: 15 symptomatic rejection, 903 asymptomatic patients and controls.	25 cp/mL or 0.09%	Rejection (cp/mL): AUC 0.68 Rejection (%): AUC 0.65 *At 25 cp/mL:* Symptomatic rejection (cp/mL): AUC 0.87; Sens 94.1%; Spec 80.7%; PPV 8.6%; NPV 99.9% *At 0.09%:* Symptomatic rejection (%): AUC 0.75; Sens 88.2%, 49.3%; PPV 3.1%; NPV 99.6%	Utilizing ddPCR, demonstrating its feasibility to quantify dd-cfDNA in heart tx (elaboration of earlier Böhmer et al., 2023). Absolute dd−cfDNA (cp/mL) vs relative donor fraction percentage, where absolute levels performed better. Samples >14d post-heart tx.	(23)
Lung					
De Vlaminck et al., 2015 SNP-based shotgun seq	Single center prospective study with 51 pts and 398 serial plasma samples; 108 TBBx. In performance: 85 quiescence (A0), 13 minimal (A1), 9 mild (A2), 7 mod/sev (A3/A4)	1%	AR (≥A3): AUC 0.90; Sens 100%; Spec 73% AR (A2-A4): AUC 0.76 AR (A1-A4): AUC 0.70	Early study demonstrating kinetics and utility of dd-cfDNA as tool for lung tx surveillance. Correlation dd-cfDNA levels and transplant tissue mass. Samples before d60 excluded for test performance analysis.	(24)
Agbor-Enoh et al., 2019 SNP-based shotgun seq	Multicenter prospective study with focus on allograft failure and mortality with 106 pts; 46 developed CLAD or died from respiratory causes. 1145 plasma samples.	N/A	Average levels of dd-cfDNA over first 3 mo were 3.6%, 1.6% and 0.7% for the upper, middle, and low tertiles. Pts in upper tertile had a 6.6-fold higher risk (p=0.007) of developing allograft failure (death, re-tx or CLAD) compared to pts in low and middle tertiles.	Focus on ability of dd-cfDNA to predict allograft failure/mortality. Demonstrating higher early dd-cfDNA levels strongly predict future allograft failure (CLAD) and death, often preceding functional deterioration.	(21)
Jang et al., 2021 SNP-based shotgun seq	Multicenter prospective study with 148 pts, longitudinal sampling; 87 AR episodes (7 ACR 1 with allograft dysfunction, 23 ACR ≥2 with + w/o allograft dysfunction and 57 clinical AMR). 484 biopsies; 377 no abnormal findings, 79 ACR. 1549 plasma samples	0.5% and 1%	AR: AUC 0.89; AMR: AUC 0.93; ACR: AUC 0.83 *At 0.5% cut-off:* AR: Sens 95%; Spec 65%, PPV 51%; NPV 96% AMR: Sens 100%; Spec 65% ACR: Sens 85%; Spec 65% *At 1% cut-off:* AR: Sens 77%; Spec 84%, PPV 64%; NPV 90% AMR: Sens 89%; Spec 84% ACR: Sens 55%; Spec 84%	Providing evidence that dd-cfDNA may be superior to histopathology at monitoring for lung allograft injury and detecting AR. Dd-cfDNA levels rose six-fold higher in AR episodes compared to control episodes. Dd-cfDNA levels also correlated with severity of lung function decline and histological grading of rejection.	(25)
Rosenheck et al., 2022 NGS; Prospera Lung (Natera)	Single-center prospective study with 103 pts, 195 samples (99 stable and 35 AR; 27 ACR, 8 AMR)	1%	AR vs stable: AUC 0.91; Sens 89.1%; Spec 82.9%; PPV 51.9%; NPV 97.3% Combined allograft injury (AR + CLAD/NRAD + infection): AUC 0.76; Sens 59.9%; Spec 83.9%; PPV 43.6%; NPV 91.0%	Clinical validation of centralized assay providing evidence for detection of AR and other allograft injury in lung tx pts.	(26)
Liver					
Schütz et al., 2017 ddPCR	Multicenter prospective study with 107 pts; 17 BPAR (31 samples), 88 stable (393 samples)	10%	AR: AUC 96.5%; Sens 90.3%; Spec 92.9% Numerous other performance evaluations, including conventional LFTs (AST, ALT, γ-GT and bilirubin)	Early study showing kinetics and utility of dd-cfDNA by ddPCR, allowing for earlier and more sensitive discrimination of AR in liver tx pts as compared with conventional LFTs.	(27)
Levitsky et al., 2022 NGS	Multicenter prospective study with 219 pts; 57 AR, 68 ADNR, 94 with normal function phenotype (referred to as “TX” in the study)	5.3% (AR vs TX); 20.4% (AR vs ADNR); 15.0% (AR vs no-AR)	AR vs. normal function (*cut-off 5.3%*): AUC 0.95; Sens 100%; Spec 80%; PPV 62.5%, NPV 100% AR vs. ADNR (*cut-off 20.4%*): AUC 0.71; Sens 75%; Spec 60%; PPV 38.5%; NPV 87.8% AR vs no-AR (all other phenotypes, *cut-off 15.0%*): AUC 0.85; Sens 87.5%; Spec 80%; PPV 58.3%; NPV 95.1%	Different cut-offs for different categories established from data. Demonstrated elevations of dd-cfDNA prior to conventional liver tests, as well as reduction of dd-cfDNA following AR treatment.	(28)

ACR, acute cellular rejection (also referred to as TCMR, T-cell mediated rejection); ADNR, acute dysfunction no rejection; AMR, antibody-mediated rejection (also referred to as ABMR); AR, acute rejection; AUC, area under the curve analysis; BPR, biopsy-proven rejection; CLAD/NRAD, chronic lung allograft dysfunction/neutrophilic-responsive allograft dysfunction; ddPCR, droplet digital PCR; EMB, endomyocardial biopsy; LDT, laboratory developed test (also known as centralized); LFTs, liver function tests; MMDx, molecular microscope diagnostic system; NPV/PPV, negative/positive predictive value; Rej, rejection; Sens, sensitivity; SOC, standard of care; Spec, specificity; TBBx, transbronchial biopsy; Tx, transplantation.

## 2 Technological evolution in dd-cfDNA quantification

### 2.1 Early approaches: qPCR and short tandem repeat analysis

Initial efforts included quantifying dd-cfDNA using real-time PCR (qPCR) and short tandem repeat (STR) analyses. qPCR targeted genetic differences, such as Y-chromosome sequences in sex-mismatched transplants or small SNP/indel panels, requiring prior genotyping of the donor and recipient (7, 29). These assays are accessible and relatively inexpensive but are limited in their applicability and precision, particularly for detecting very low dd-cfDNA fractions (2). STR analysis, adapted from forensic and chimerism testing, also enabled dd-cfDNA estimation but was labor-intensive, less sensitive at low levels, and required informative markers identified through previous genotyping. While valuable for proof-of-concept, these methods lack the robustness needed for routine transplant surveillance.

### 2.2 Next-generation sequencing: comprehensive profiling and broad applicability

Next-Generation Sequencing (NGS) has transformed dd-cfDNA analysis by enabling highly sensitive discrimination of donor-derived fragments using targeted or genome-wide approaches suitable for scalable, high-throughput testing (30, 31). SNP- and indel-based panels further enhance analytical sensitivity, particularly at low fractions, and several commercial assays have been widely adopted in clinical practice (**Table 2**). These include centralized assays, such as AlloSure (CareDx) and Prospera (Natera), and decentralized assays, such as One Lambda Devyser Accept cfDNA (Devyser) and AlloSeq (CareDx) (3). The main challenges to date include high costs, extended turnaround times for centralized processing, and implementation considerations for decentralized assays. Nevertheless, NGS offers a comprehensive and analytically powerful technology for dd-cfDNA monitoring, setting a standard for clinical adoption in both centralized and de-centralized settings.

**Table 2:** Commercially available donor-derived cell-free DNA (dd-cfDNA) assays for monitoring after solid organ transplantation. The table highlights key inter-assay differences relevant to clinical implementation and interpretation.

Assay/service name	Provider	Analytical methodology	Marker strategy	Testing model	Key supporting study
AlloSure^®^	CareDx	NGS; relative quantification (% dd-cfDNA)	405 SNPs	Centralized	(14)
AlloSeq^®^				Decentralized	(32)
Prospera™	Natera	NGS; relative quantification (% dd-cfDNA)	13–392 SNPs	Centralized	(10)
TRAC™	Viracor Eurofins	NGS (genome-wide); relative quantification (% dd-cfDNA)	>100–000 SNPs	Centralized	(33)
AlloNext™	Eurofins Genome	NGS; relative quantification (% dd-cfDNA)	>500 SNPs	Centralized	(34)
One Lambda™ Devyser Accept cfDNA	Devyser	NGS; relative quantification (% dd-cfDNA)	50 indels	Decentralized	(19)
GraftAssureCore™ (previously VitaGraft)	iMDx (previously Oncocyte)	ddPCR; relative and absolute quantification (% dd-cfDNA and cp/mL)	45 SNPs	Centralized	(15, 27)
GraftAssureDx™				Decentralized	(35)
HoloGRAFT ONE™	Omixon	dPCR; relative and absolute quantification (% dd-cfDNA and cp/mL)	43 CNVs	Decentralized	(36)

NGS, next-generation sequencing; dd-cfDNA, donor-derived cell-free DNA; ddPCR, droplet digital PCR; SNP, single nucleotide polymorphism; indel, insertion/deletion; dPCR, digital PCR; CNV, copy number variant.

### 2.3 Digital PCR: enhanced precision and absolute quantification

Although commercial dd-cfDNA testing has been dominated by next-generation sequencing workflows, non-NGS solutions based on digital PCR (dPCR) are also available. dPCR comprise partition-based techniques for nucleic-acid quantification that offer enhanced precision and sensitivity in detecting low-abundance targets (2, 3). The reaction mixture is divided into many discrete partitions containing zero or a few template molecules. Following PCR amplification, each partition is identified as either positive or negative for the target sequence, and the absolute target concentration is inferred from the fraction of positive partitions using Poisson statistics. This eliminates the need for standard curves and reduces the impact of amplification-efficiency variations compared to qPCR. Partitioning can be achieved using droplet emulsions, microwell or chip-based arrays, and other microfluidic formats (37). This technology is used in commercial dd-cfDNA solutions, including GraftAssure (iMDx) and HoloGRAFT ONE (Omixon) (2, 35, 36, 38) (**Table 2**). Beyond commercial kits, non-commercial dPCR strategies can quantify dd-cfDNA using informative donor–recipient HLA mismatches (e.g., HLA-DRB1), enabling rapid and low-infrastructure testing in selected settings (39). Limitations of dPCR include the finite number of partitions, which constrains dynamic range and multiplexing potential, challenges in scaling throughput, and platform-specific technical variability such as variability in droplet generation for droplet digital PCR or loading and partition-volume effects in chip-based systems (40, 41).

## 3 Clinical utility of dd-cfDNA and evidence across organ types

The primary aim of any transplant monitoring tool is to improve long-term graft survival and patient outcomes by enabling earlier and more accurate detection of allograft injury. Traditional functional markers such as serum creatinine, estimated glomerular filtration rate (eGFR), liver transaminases, or pulmonary function tests (PFTs) are widely available and inexpensive, but they are typically late and non-specific indicators of damage; abnormal values may reflect advanced or irreversible injury, and modest changes can occur even in patients with stable graft function. Allograft biopsy remains the gold standard for diagnosing rejection and other forms of graft pathology. Histological assessment enables the direct visualization of cellular infiltrates, tissue damage, and antibody deposition, offering essential insights for guiding treatment decisions. However, biopsies are invasive procedures that carry the potential risk of complications, including bleeding, infection, pain, and, in rare cases, organ damage. Beyond procedural risks, biopsies are prone to sampling errors, as rejection can be focal, and a small tissue sample may not accurately represent the condition of the entire organ. Additionally, there is inter-observer variability among pathologists when interpreting biopsy findings, particularly in borderline or ambiguous cases. Moreover, the cost and logistical demands of performing biopsies, particularly in routine surveillance protocols, are high. Many centers have reduced the frequency of surveillance biopsies because of these limitations.

In contrast, donor-derived cell-free DNA (dd-cfDNA) provides a direct, quantitative measure of allograft injury by capturing the release of donor-derived nucleic acids into the recipient’s circulation, often preceding evident clinical or biochemical signs of deterioration. Several observational cohort and registry-based studies have shown that persistently elevated dd-cfDNA levels are associated with biopsy-proven rejection, the development of *de novo* donor-specific antibodies, accelerated decline in graft function, and worse long-term graft survival (**Table 1**). In many of these studies, low dd-cfDNA levels were characterized by a high negative predictive value for active rejection, supporting the use of dd-cfDNA as a rule-out tool in routine surveillance, whereas rising or persistently elevated levels identified patients at increased risk who may benefit from closer monitoring or diagnostic biopsy. Risk stratification based on dd-cfDNA may also guide individualized immunosuppressive strategies, thereby reducing the likelihood of graft failure in the future. Pediatric transplant recipients, who have more potential years at risk, may especially benefit from improved monitoring strategies to increase long-term graft preservation. For example, data from the Diagnosing Acute Rejection in Kidney Transplant Recipients (DART) and Donor-Derived Cell-Free DNA Outcomes AlloMap Registry (D-OAR) studies support the prognostic value of dd-cfDNA and its potential to reduce biopsy frequency in clinically stable kidney and heart transplant patients, respectively (11, 14). These associations have been reported across multiple organ types and clinical contexts, including asymptomatic surveillance, evaluation of graft dysfunction, and follow-up after intensified immunosuppression or rejection treatment (7, 13, 42, 43).

Immunological monitoring, such as detection of donor-specific anti-human leukocyte antigen antibodies (DSA), plays a role, particularly in identifying patients at risk for ABMR, a leading cause of late graft loss. However, the mere presence of DSAs does not always correlate with active graft injury, as they can also be found in patients with stable graft function (44). Research has shown that combining dd-cfDNA with DSA may enhance diagnostic accuracy, thereby facilitating the early identification and treatment of ABMR (45). Recently, Akifova et al. reported the results of a prospective, single-center, open-label randomized clinical trial (NCT04897438) to evaluate the clinical benefit of dd-cfDNA-guided biopsy for the early detection of ABMR in kidney transplant recipients with *de novo* donor-specific anti-human leukocyte antigen antibodies (46). Forty patients were randomized to either a dd-cfDNA-guided biopsy group or a clinician-guided biopsy group and monitored over 12 months with serial dd-cfDNA assessments at 1, 3, 6, 9, and 12 months. A dd-cfDNA threshold of >50 cp/mL (determined by digital PCR) prompted biopsy in the intervention group. Among the 39 patients with functioning grafts at study completion, ABMR was diagnosed significantly earlier in the dd-cfDNA-guided group than in the control group (median 2.8 vs. 14.5 months, p=0.003). Longitudinal dd-cfDNA monitoring demonstrated a positive predictive value (PPV) of 77% and an NPV of 85% for ABMR detection. This study provides randomized clinical evidence supporting that dd-cfDNA-guided surveillance can facilitate the early diagnosis of ABMR and timely initiation of therapy in kidney transplant recipients, contributing to the growing body of evidence for the clinical utility of dd-cfDNA testing in post-transplant monitoring.

Currently, randomized controlled trials that test dd-cfDNA-guided management strategies with hard endpoints, such as graft survival or mortality, are still limited. However, the consistent correlation between dd-cfDNA elevation, histologic injury, and adverse outcomes, along with its ability to detect allograft damage earlier than traditional biomarkers, strongly supports its clinical utility as part of a multimodal monitoring strategy. In the following sections, we summarize the organ-specific evidence for dd-cfDNA in kidney, heart, lung, and liver transplantation, highlighting the similarities and differences in diagnostic performance, prognostic value, and integration into existing surveillance workflows.

### 3.1 dd-cfDNA as a non-invasive biomarker for kidney allograft monitoring

In the past, kidney allograft surveillance relied on serum creatinine levels and protocol or for-cause biopsies. However, serum creatinine is an insensitive and late marker of rejection, often increasing only after significant graft damage has occurred. Such elevations can also result from non-rejection causes, such as drug nephrotoxicity and dehydration (47). Numerous studies have demonstrated that elevated levels of dd-cfDNA in the plasma of kidney recipients are associated with acute rejection, including TCMR and AMR (10, 48). For example, a study using the NGS-based Prospera assay (provided by Natera) found significantly higher median dd-cfDNA levels in samples with biopsy-proven active rejection (2.3%) than in those with borderline rejection (0.6%), other injury (0.7%), and stable allografts (0.4%). At a cutoff of >1% dd-cfDNA, this assay showed 88.7% sensitivity and 72.6% specificity for discriminating active rejection from non-rejection status, with an AUC of 0.87 (10).

A key advantage of dd-cfDNA is its potential to detect subclinical rejection, that is, rejection occurring in the absence of overt graft dysfunction (49). In kidney transplantation studies, dd-cfDNA levels can increase weeks to months before histological evidence of graft rejection or changes in serum creatinine, providing a valuable window for intervention (1, 50). Furthermore, dd-cfDNA has a high NPV; low dd-cfDNA levels (e.g., <0.5% or <1%, depending on the assay and clinical context) can confidently rule out active rejection, potentially helping to avoid unnecessary biopsies in stable patients or those with non-rejection causes of serum creatinine elevation (10, 48).

More recently, studies have explored the combined use of dd-cfDNA with peripheral blood gene-expression profiling (GEP) to refine diagnostic performance. In a *post-hoc* analysis of paired surveillance biopsies from stable kidney transplant recipients, Park et al. found that each assay alone provided similar discrimination for subclinical rejection (AUC 0.75 for GEP and 0.72 for dd-cfDNA) (33). Concordant results improved clinical interpretability, with a negative predictive value of 88% when both tests were negative and a positive predictive value of 81% when both were positive. A combined model using multivariable logistic regression increased discrimination further, achieving an AUC of 0.81 and 0.76 on external validation. Notably, the assays appeared complementary by rejection phenotype, with GEP performing better for cellular rejection and dd-cfDNA performing better for antibody-mediated rejection. In contrast, a recent real-world study by Sellarés et al. evaluated this combined biomarker strategy in routine practice and highlighted the need for independent validation (51). While the combined use of dd-cfDNA and GEP did not improve rejection diagnosis across all settings, the study found dd-cfDNA useful for detecting AMR or mixed rejection when interpreted with donor-specific antibodies in patients with graft dysfunction. This work cautioned against broad generalization of combined testing, noting that biomarker performance depends on patient risk profile, timing post-transplant, and rejection probability. Collectively, these findings highlight the need for prospective trials to define optimal clinical application of dd-cfDNA–based biomarkers in kidney transplantation.

While dd-cfDNA is a strong indicator of allograft injury, it is not entirely specific for rejection, as other conditions such as acute tubular necrosis, BK virus nephropathy, and severe pyelonephritis can also cause elevated levels (47, 52). Therefore, dd-cfDNA results are best interpreted in conjunction with other clinical and laboratory data to refine the diagnosis and guide management. Reflecting its growing clinical utility, the American Society of Transplant Surgeons (ASTS) has recommended dd-cfDNA testing in adult kidney transplant recipients to monitor rejection as a component of post-transplant surveillance (American Society of Transplant Surgeons, 2024). Concurrently, a recent consensus statement from the European Society of Organ Transplantation (ESOT) offers an evidence-based framework for the clinical application of molecular biomarkers, including dd-cfDNA, in kidney transplantation (53). Using PICO questions, systematic literature review, and GRADE ratings, the ESOT statement consolidates available evidence and explicitly addresses the context of use, strength of recommendations, and current limitations of dd-cfDNA testing. The statement notably emphasizes that dd-cfDNA is most effectively used as an adjunctive tool in specific clinical scenarios, such as ruling out rejection, particularly antibody-mediated rejection, when evaluating graft dysfunction, rather than serving as a standalone or universal surveillance test. It also highlights the necessity for further prospective interventional studies to establish outcome benefits. Collectively, these recommendations advocate a balanced, evidence-based approach to dd-cfDNA implementation and emphasize the importance of context-driven, appropriate use in routine practice.

### 3.2 dd-cfDNA in heart transplant rejection: comparison with endomyocardial biopsy and echocardiography

Surveillance for acute rejection in heart transplant recipients has historically relied heavily on endomyocardial biopsy (EMB), an invasive procedure associated with procedural risks, including cardiac perforation, valve injury, arrhythmias, and vascular complications (11). The frequency of EMB varies considerably depending on the country, medical center, patient age, and risk profile. Nonetheless, it is generally recommended for surveillance during the initial 3–12 months post-transplantation and for high-risk cases thereafter (54). It is not uncommon to schedule weekly biopsies during the first month, followed by biweekly procedures in the second and third months, and subsequently monthly from the fourth to the sixth month, resulting in a substantial total number of EMBs. Echocardiography provides a functional assessment of the graft, but changes in echocardiographic parameters are often late and non-specific indicators of rejection. Dd-cfDNA is a non-invasive biomarker for cardiac allograft injury, with the potential to reduce the reliance on surveillance EMBs (7, 11). In stable heart transplant recipients, dd-cfDNA levels are typically very low, often <0.2% (2). Elevations above baseline dd-cfDNA are associated with allograft injury, including acute cellular rejection (ACR grade ≥2R) and antibody-mediated rejection (AMR pAMR ≥1). In the prospective multicenter D-OAR study evaluating the AlloSure dd-cfDNA assay (CareDx) in heart transplant recipients, median dd-cfDNA was higher in acute rejection than in no rejection (0.17% vs 0.07%). At a 0.2% threshold, dd-cfDNA demonstrated 44% sensitivity and a 97% negative predictive value (11). The high NPV suggests that low dd-cfDNA levels can reliably exclude significant rejection and may allow the deferral of EMB.

Several studies have compared the performance of dd-cfDNA and EMB (**Table 1**). The lower threshold observed in heart transplants compared to other organ types underscores the necessity for assays with high sensitivity and precision. Notably, in heart transplants, dd-cfDNA levels can increase several weeks before clinical or histological evidence of rejection, providing an opportunity for earlier intervention. For instance, one ddPCR study reported significant increases in dd-cfDNA 9–30 days prior to biopsy-confirmed rejection. Integrating dd-cfDNA with other non-invasive biomarkers, such as GEP, has been demonstrated to enhance surveillance accuracy and risk stratification (55). While dd-cfDNA is a strong indicator of myocardial injury, it may not consistently differentiate rejection from other causes, such as ischemia or myocarditis (56). Nonetheless, dd-cfDNA is increasingly being incorporated into clinical practice, facilitating earlier detection, supporting personalized surveillance, and reducing the burden of EMB (11).

### 3.3 Lung allograft management: addressing diagnostic challenges and improving outcomes with dd-cfDNA

Complications after lung transplantation remain diagnostically challenging and include acute cellular and antibody-mediated rejection, infections, and the development of chronic lung allograft dysfunction (CLAD), which remains the leading cause of late mortality after transplantation (57, 58). Traditionally, surveillance relies on PFTs, imaging, and invasive procedures, such as transbronchial biopsy (TBBx) and bronchoalveolar lavage (BAL). However, PFTs are often late indicators of dysfunction, whereas TBBx carries procedural risks and has limited sensitivity. In this setting, dd-cfDNA is emerging as a promising non-invasive biomarker of allograft injury that complements standard testing. Elevations above the baseline have been associated with ACR, AMR, and CLAD development. In one targeted NGS study, dd-cfDNA levels were significantly higher in patients with rejection than in stable controls. At a 0.85% threshold for combined rejection, the sensitivity was 55.6%, specificity was 75.8%, and NPV was 83.6% (12). These findings suggest that dd-cfDNA may serve as a rule-out tool in a multi-marker strategy.

Some studies have reported no significant difference in dd-cfDNA levels between infection cases and stable controls, indicating its potential utility in differentiating rejection from infection. However, infections that result in substantial tissue damage may still lead to elevated dd-cfDNA levels (26, 59). As observed with other organ types previously discussed, a high NPV is particularly useful, as low dd-cfDNA levels may reduce dependence on TBBx. Research on dd-cfDNA in BAL has also shown promise; one study demonstrated that BAL cfDNA, when combined with CXCL10, can detect and differentiate between CLAD subtypes and serve as a predictor of graft survival (60).

Integrating dd-cfDNA into lung transplant management could enable earlier recognition of rejection, aid in differentiation from infection, and guide individualized surveillance protocols. Larger prospective studies are needed to establish optimal thresholds, validate performance across diverse scenarios and confirm cost-effectiveness. Given the complexity of post-transplant care, dd-cfDNA should always be interpreted in conjunction with clinical findings, imaging, and microbiological data to maximize its diagnostic value. As lung-specific thresholds and multimodal panels mature, dd-cfDNA-guided surveillance has the potential to shift practice toward more personalized, risk-adapted care while safeguarding against overtreatment in this complex population.

### 3.4 Liver allograft surveillance: early detection of graft dysfunction and rejection pathways using dd-cfDNA

Traditionally, postoperative monitoring after liver transplantation relies on liver function tests (LFTs), such as alanine aminotransferase (ALT), aspartate aminotransferase (AST), and bilirubin. While useful, these markers lack specificity for the underlying cause of injury and often increase only after significant graft dysfunction. Liver biopsy remains the gold standard for diagnosing rejection, but it is invasive, costly, and unsuitable for frequent surveillance. In recent years, dd-cfDNA has shown promise as a non-invasive biomarker for the early detection of allograft injury and rejection in the context of liver transplantation (61). Immediately post-transplantation, dd-cfDNA levels can approach 90% of the total cfDNA, reflecting ischemia-reperfusion injury and transplanted organ mass. In uncomplicated cases, levels decline within 7–10 days to below 10–15% and subsequently stabilize at a lower baseline. Deviations from this expected decline may have prognostic value (30, 62). Elevations above the baseline may indicate rejection, biliary complications, vascular issues, or recurrent disease. In a ddPCR study of 115 recipients, Schutz et al. reported that a 10% dd-cfDNA threshold achieved 90% sensitivity and 93% specificity for rejection, with an AUC of 0.97, outperforming same-day LFTs (27).

Importantly, dd-cfDNA levels can increase before abnormal LFTs results, enabling earlier diagnosis. Longitudinal monitoring also facilitates the establishment of individual baselines and the detection of clinically meaningful deviations. Therefore, integrating dd-cfDNA into routine follow-up may reduce unnecessary biopsies, improve diagnostic accuracy, and support personalized management strategies. Ongoing research aims to refine thresholds, validate dd-cfDNA across diverse patient populations, and explore its role in characterizing different rejection phenotypes.

## 4 Centralized versus decentralized testing models: implications for turnaround, access, and workflow

Centralized dd-cfDNA testing, available by numerous providers (**Table 2**), requires shipping blood or plasma samples to specialized reference laboratories, where cfDNA extraction, sequencing, and bioinformatic analyses are conducted under standardized conditions under expert supervision (32). Providers typically supply collection kits, prepaid shipping, and electronic reporting to streamline logistics. This model ensures consistency, high-quality control, and economies of scale. However, shipping and batch processing extend turnaround times to 3–7 days or longer, limiting their utility in urgent clinical settings, such as suspected acute rejection (63). Centralized services also incur high costs and create dependence on a single provider.

Decentralized testing enables local or regional laboratories to perform dd-cfDNA analysis in-house using standardized kits on existing ddPCR or NGS platforms (19, 64). By eliminating transport delays, the results can be returned within one day, supporting faster clinical decision-making. Decentralization also enhances accessibility and facilitates integration with local information systems, fostering closer collaboration between clinicians and laboratories. Kits such as One Lambda Accept cfDNA (Devyser) and AlloSeq cfDNA (CareDx) have demonstrated strong concordance with centralized testing (19, 32). Challenges include the need for laboratory expertise, validation, bioinformatics infrastructure, and quality control systems to ensure consistency between laboratories.

Decentralization involves initial investments, such as acquiring equipment, hiring staff with molecular genomics expertise, and assuming full validation and routine QA/QC responsibilities. These factors may limit its adoption in centers with low patient volume. Because NGS workflows are most cost-effective when conducted in batches, low-throughput programs might encounter unfavorable per-test economics despite potential improvements in turnaround time. Therefore, formal economic evaluations comparing centralized and decentralized models are still necessary. In the short term, a mixed ecosystem is likely to emerge: centralized send-out testing will remain appealing for smaller or lower-volume programs, whereas large transplant centers may favor decentralized workflows to achieve approximately 24-hour analytical turnaround times and better integration with local care pathways, as supported by recent multicenter equivalence data. However, robust clinical validation and multicenter harmonization are crucial before widespread adoption. Looking ahead, true point-of-care dd-cfDNA testing remains an aspiration; emerging nucleic-acid detection platforms, such as nanopore and microfluidic biosensors (65, 66), could eventually facilitate clinic- or home-based monitoring. However, current evidence does not support its routine use. Overall, the choice between centralized and decentralized models is context dependent and dynamic. Ongoing comparative effectiveness and cost-effectiveness studies, along with external quality programs, will be vital in determining the value of each model.

## 5 Discussion

### 5.1 Clinical utility and prognostic value of dd-cfDNA in post-transplant surveillance

dd-cfDNA may provide a non-invasive adjunct to post-transplant monitoring, complementing traditional markers that are less sensitive and often increase only after significant graft damage (7, 43). A major strength of this test is its high negative predictive value (often >90%), which allows clinicians to confidently rule out rejection and potentially reduce unnecessary biopsies. Beyond single time-point assessments, longitudinal monitoring of dd-cfDNA can be valuable, as dynamic changes in levels may indicate evolving graft injury, predict impending rejection, reflect treatment response, and thus further support individualized patient management over time.

Sensitivity appears to be highest for ABMR, whereas it is lower for T cell–mediated forms of rejection (43, 67). Notably, persistently elevated dd-cfDNA levels have been associated with long-term allograft injury, dnDSA development, and a greater risk of eGFR decline in kidney transplant recipients (17). However, most evidence supporting these associations derives from observational cohorts, registries, and retrospective single-center studies. Randomized, prospective interventional trials focusing on specific clinical outcomes, such as graft survival and mortality, remain relatively rare. This scarcity limits the ability to draw causal inferences about whether dd-cfDNA-guided monitoring directly enhances these outcomes. Emerging interventional evidence, including findings from the prospective randomized study by Akifova et al., supports the feasibility of dd-cfDNA-guided management pathways. However, this study and others like it are not designed to determine whether dd-cfDNA-guided interventions improve long-term outcomes. Therefore, additional prospective, ideally multicenter interventional trials are necessary to establish the impact of protocolized dd-cfDNA-guided strategies on clinically meaningful endpoints.

While dd-cfDNA is a useful adjunct biomarker of allograft injury, current evidence most strongly supports its use in defined clinical contexts where it is likely to add incremental value relative to standard-of-care monitoring. In particular, dd-cfDNA appears most actionable when used to (i) triage patients with graft dysfunction and support decisions regarding diagnostic biopsy; (ii) refine risk assessment in patients with donor-specific antibodies, where elevations may increase suspicion for antibody-mediated injury and prompt timely evaluation; and (iii) support risk-stratified surveillance in higher-risk periods or patients (e.g., early post-transplant, prior rejection, intensified immunosuppression), where trends may help identify evolving injury and guide follow-up intensity. In contrast, evidence remains less robust for routine, universal high-frequency testing in low-risk stable recipients and for using dd-cfDNA as a stand-alone decision tool. Given the cost and operational requirements, its clinical utility is likely greatest when used selectively, particularly when rejection is clinically plausible and when results are expected to inform near-term decisions such as biopsy triage or treatment escalation.

### 5.2 Challenges and barriers to broader adoption

Several technical and interpretative complexities impede broader clinical adoption, including methodological differences between commercially available assays. For example, while AlloSure and Prospera both utilize SNP-based NGS, they differ in the number of targeted SNPs (e.g., AlloSure initially 266, now 405; Prospera 13,392) (31), while Devyser One Lambda Accept cfDNA utilizes 50 indels (68). Variations in dd-cfDNA measurements across different platforms necessitate the consistent use of a single method for longitudinal patient monitoring (63, 69). Additionally, standardizing sample collection, handling, processing, and storage is crucial, as these factors can significantly influence cfDNA levels and their characteristics. Interpretative challenges arise from the non-specific nature of dd-cfDNA in detecting rejection; elevated levels may indicate various types of allograft injury, such as infection or surgical complications (47, 67). Identifying the exact cause of elevation necessitates the integration of other clinical data and, at times, remains elusive (7).

Inter-assay comparability and interchangeability remain not fully established, and dd-cfDNA results should not be assumed to be directly transferable across different platforms. Comparability may involve agreement in numerical values, concordance of clinical classification at predefined cutoffs, and the preservation of longitudinal trends when transitioning between assays. These aspects may vary due to differences in marker panels, chemistry, bioinformatic modeling, and reporting formats among assays. In the context of kidney transplantation, paired-sample comparisons of two widely used commercial NGS assays (AlloSure and Prospera) have shown broadly similar discrimination but revealed that agreement can be cutoff-dependent, with discordance observed when applying manufacturer-recommended thresholds (69). In heart transplantation, retrospective paired testing similarly found substantial agreement between AlloSure and Prospera, while emphasizing that the choice of thresholds can influence concordance (70). Method-focused cross-technology comparisons further suggest that fractional dd-cfDNA (%) may be more comparable across analytical approaches than absolute quantification, whereas absolute copy-number metrics can exhibit proportional bias across methods (71). Finally, a large multicenter study comparing a decentralized NGS kit (AlloSeq cfDNA) with a centralized service (AlloSure) reported high correlation but systematic differences, highlighting the necessity for bridging studies before applying cross-assay thresholds or switching platforms in longitudinal monitoring (32). Overall, the number of published head-to-head comparisons remains limited, and larger prospective bridging studies are needed to determine when assays can be used interchangeably and how thresholds should be harmonized across platforms.

Another important source of inter-assay differences is variation in workflow design. Across both NGS- and dPCR-based dd-cfDNA quantification methods, approaches differ in their reliance on direct donor or recipient genotyping to identify informative loci; while some require explicit genotyping, others leverage bioinformatic inference from population allele frequencies and mixed-sample modeling to estimate donor contribution without prior donor genotyping. In addition, the two technologies differ in how relative and absolute quantification are generated. Most NGS-based assays primarily report fractional dd-cfDNA (%), even though absolute counts can be bioinformatically inferred, whereas digital PCR–based assays directly measure absolute dd-cfDNA (copies/mL, cp/mL) at the assay level.

The accessibility and cost of dd-cfDNA testing may present additional challenges to its widespread use. Detailed insights into its long-term cost-effectiveness, both overall and within various patient sub-groups, are essential (67, 72). The economic burden on healthcare systems and individual patients necessitates a thorough analysis, particularly in terms of long-term surveillance. Geographic disparities in access to specialized testing facilities may limit the availability of this method in certain regions. The logistics of sample collection, especially for patients in remote areas, require a robust infrastructure. During the COVID-19 pandemic, the combination of remote home phlebotomy and dd-cfDNA analysis proved to be a viable model for maintaining surveillance, highlighting a potential pathway for improved accessibility (73). Nonetheless, comprehensive cost-benefit analyses that incorporate factors such as reduced biopsy rates and improved long-term graft outcomes are crucial for the widespread adoption of this approach (67).

### 5.3 Future directions and translational outlook

Optimizing the clinical integration of dd-cfDNA will require progress in several areas. Current experience highlights substantial heterogeneity in pre-analytical handling, assay design, and reporting between laboratories and commercial platforms, which limits cross-study comparability and complicates the definition of universal thresholds (42, 74, 75). Therefore, the development of standardized guidance and consensus protocols for analytical performance, quality control, reporting units, and interpretation frameworks is essential to ensure reproducibility and support broader regulatory and guideline adoption of dd-cfDNA-based monitoring. Defining optimal testing intervals, particularly in the early post-transplant period when the risk of rejection is highest, will also help guide clinical use (43, 76). The integration of dd-cfDNA with DSA testing, traditional functional markers, and new emerging biomarkers could improve diagnostic specificity and enable multimodal surveillance strategies. In parallel, ongoing educational initiatives are needed to familiarize clinicians with appropriate test use and interpretation (77).

Future research priorities should focus on conducting adequately powered multicenter studies to establish clinically actionable dd-cfDNA thresholds across various platforms and organ types, determine evidence-based monitoring intervals, and demonstrate that dd-cfDNA-guided management enhances graft and patient survival. Pediatric recipients, who are at a high cumulative risk of biopsy exposure and long-term graft loss, require focused research in this area. Multimodal strategies that integrate dd-cfDNA with complementary blood and urine biomarkers may offer deeper mechanistic insights and better distinguish rejection from infection and other causes of graft injury (72). Beyond post-transplant surveillance, cfDNA released from donor organs into the preservation perfusate has emerged as a promising tool for pre-implantation assessment. Studies on ex vivo lung perfusion and machine-perfused liver grafts suggest that elevated perfusate levels of nuclear and mitochondrial cfDNA are linked to markers of ischemia–reperfusion injury and early post-transplant dysfunction, indicating that cfDNA could enhance existing functional and metabolic criteria for donor selection and allocation (78–80). Integrating serial perfusate cfDNA measurements with established biomarkers, such as lactate, transaminases, and flavin mononucleotide, may enable a more dynamic and quantitative assessment of graft recovery, although standardized thresholds and prospective interventional validation remain pressing needs.

### 5.4 Conclusion

Dd-cfDNA has rapidly evolved from a promising biomarker to an increasingly integral component of post-transplantation surveillance across multiple organ types. Advances in NGS and ddPCR have enabled precise and sensitive quantification, with strong evidence for detecting rejection, guiding risk stratification, and reducing reliance on biopsies across organ types. While certain challenges remain, including assay standardization, cost-effectiveness, and improved specificity, ongoing research and technological refinements are addressing these gaps. As dd-cfDNA becomes embedded in multimodal monitoring strategies, it has the potential to transform transplant medicine by supporting earlier interventions, personalized immunosuppression, and ultimately, prolonged graft and patient survival.
